# Supplementary material for: Neuroprotective Effect of Red Sea Marine Sponge Xestospongia testudinaria Extract Using In Vitro and In Vivo Diabetic Peripheral Neuropathy Models
Source: Pharmaceuticals (Basel). 2022 Oct 24;15(11):1309. doi: 10.3390/ph15111309 (PMC9693000; doi:10.3390/ph15111309)
Supplement: Supplementary file 1 [file pharmaceuticals-15-01309-s001.zip › pharmaceuticals-1908573-SI.pdf]

Toxicity studies of IP injection of sponge extract to mice

Introduction

Before investigating the efficacy of *X. testudinaria* extract using *in vivo* mode, however, the safety of *X. testudinaria* extract must be established. There is only one previous published work was done on the oral acute toxicity for *X. testudinaria* (Lin-Fu Liang, 2014). However, in the current study, the administration of *X. testudinaria* extract by IP route of administration was chosen because there is not enough study regarding *X. testudinaria* extract pharmacokinetic. Besides, it has been reported that the serum concentrations of all exogenous antioxidant nutrients were remarkably influenced by other dietary intakes (Galan *et al.*, 2005). Therefore, to avoid any possible risk of food-drug interactions, *X. testudinaria* extract was administered IP.

Methods

Determination of the LD_{50} of the sponge extract

Three male mice weighing 25-30 g received an IP injection of 2000 mg/kg of *X. testudinaria* extract.

Acute toxicity study:

Twelve male mice weighing 25-30 g were randomly divided into four groups (3 mice each). Group 1 (3 mg/kg), Group 2 (1.5 mg/kg), Groups 3 (0.75 mg/kg) and Group 4 (3 mg/kg Settled group). All groups received single injection intraperitoneal (IP) of sponge extract. After 24hr, all mice were sacrificed to observe histological signs of toxicity. Finally, blood samples were collected for biochemical analyses.

Results

Evaluation of acute toxicity of *X. testudinaria* extract in mice

Evaluation of LD_{50} of *X. testudinaria* extract

There was no mortality reported or any toxicity signs after 24 hours of IP injection of 2000 mg/kg of *X. testudinaria* extract. Moreover, mice in the 2000 mg/kg satellite group showed normal behaviour during the 14 days post IP injection.

Evaluation of different doses of *X. testudinaria* on LFT

As shown in (Figure S1 (A and B)), there is no significant difference in ALT and AST levels with all tested doses of *X. testudinaria* extract (3 mg/kg, 1.5 mg/kg, 0.75 mg m 0.38 mg, and 3 mg/kg satellite group) compared to the control group. However, there is a slightly significant increase in GGT and ALP levels in the highest tested dose of 3 mg/kg *X. testudinaria* extract compared with the control group, (Figure S1 (C and D)).

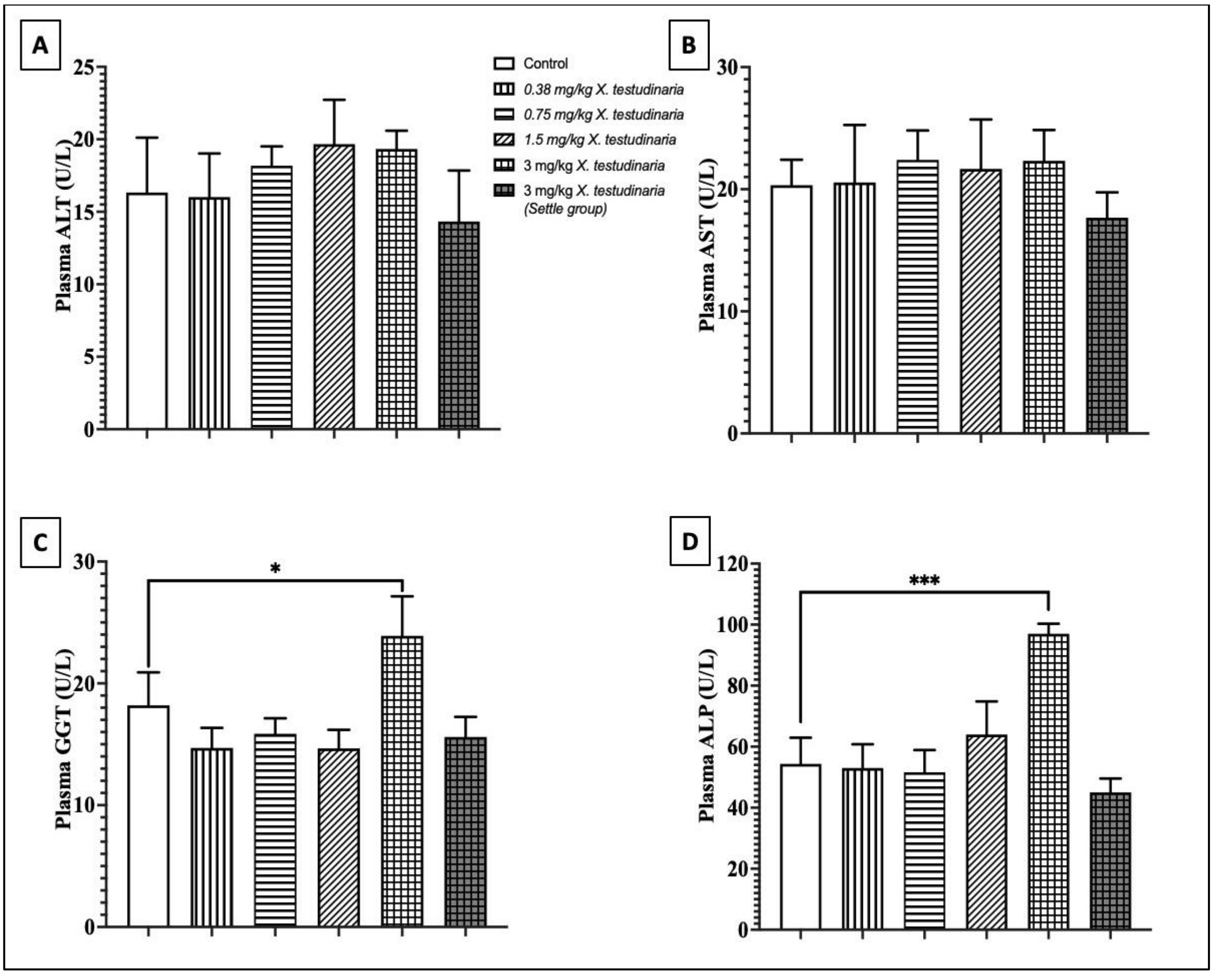

Figure S1. Evaluation of different doses of *X. testudinaria* extract on: A) Plasma alanine transaminase (ALT), B) Plasma aspartate aminotransferase (AST), C) Plasma Gamma-glutamyl transferase (GGT) and D) Plasma alkaline phosphatase (ALK). Data are presented as mean ± SD of n=3 in each group. Statistical analysis was carried out using One-Way ANOVA. *$p < 0.05$ and ***$p < 0.001$ compared with the control group.

Evaluation of different doses of *X. testudinaria* extract on liver histology.

Figure 5 shows a representative section of liver tissue from different treatment groups. The control group (Figure S2 (A)) shows the normal histology architecture of liver tissue. Administration of 0.38 mg/kg *X. testudinaria* extract did not change the histological architecture of liver tissue (Figure S2 (B)). Administration of 0.75 mg/kg of *X. testudinaria* extract showed the normal hepatocytes with mild congested central vein (Figure S2 (C)).

However, administration of 1.5 mg/kg of *X. testudinaria* extract caused some loss of normal liver architecture and vacuolated hepatocytes with dark stained nuclei and dilated central vein as shown in (Figure S2 (D)). These pathological changes were also detected with administration of the highest dose of *X. testudinaria* extract (3 mg/kg) with greater extent (Figure S2 (E)). Moreover, there is a wide separated area with degenerated hepatocytes with extravasated blood cells RBCs and inflammatory cells around degenerated bile duct. However, the normal histopathological changes were preserved in (3 mg/kg) satellite group compared to normal group as shown in (Figure S2 (F)).

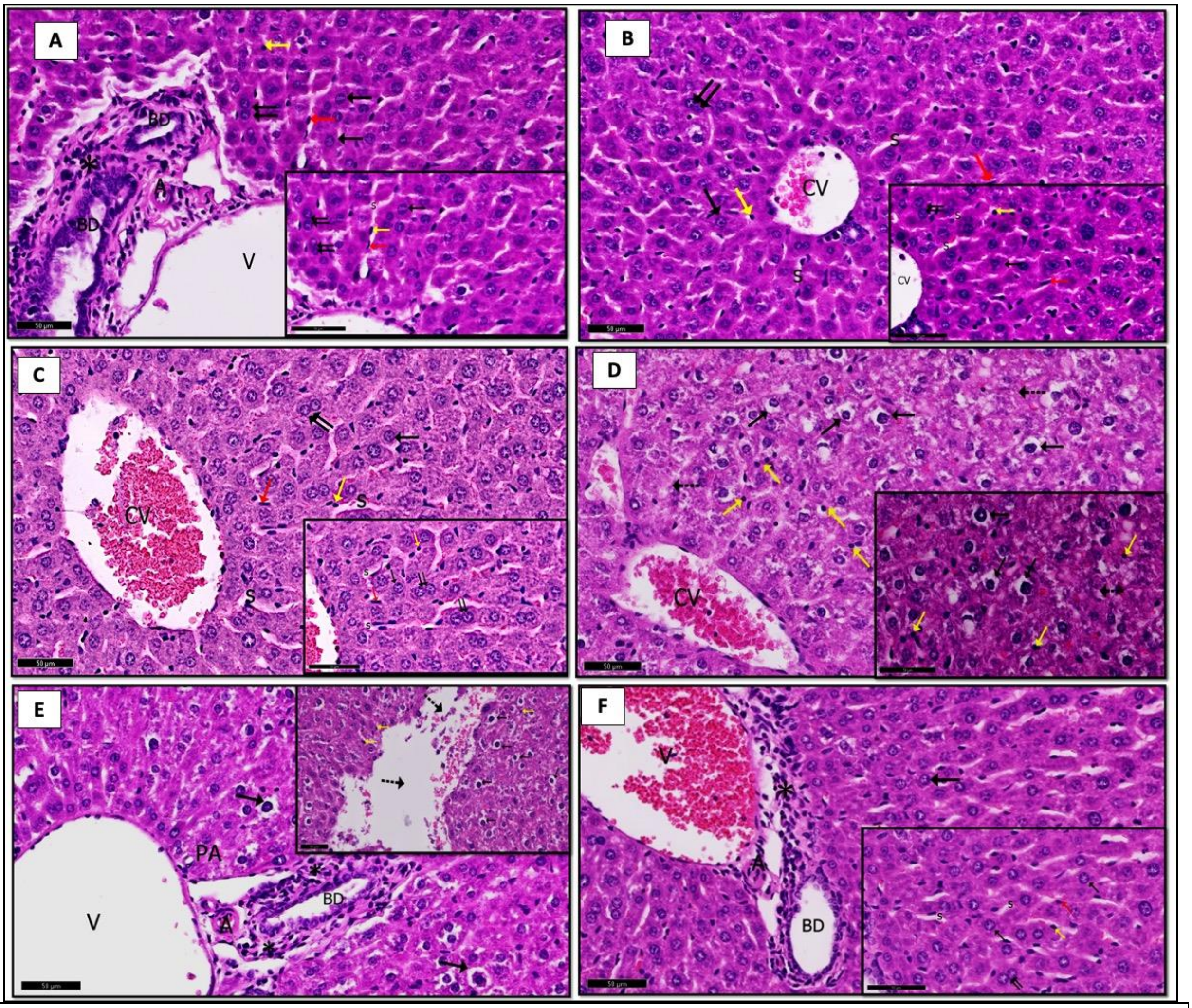

Figure S2. Effect of different doses of *X. testudinaria* extract on liver histology. A) control group with normal portal vein (V). Hepatic cords and separated from each other by hepatic sinusoids (S) lined by thin flat endothelial cells (red ↑) and Von kupffer cells (yellow↑). Note hepatocytes (black↑) with vesicular nuclei. Some hepatocytes contain two nuclei (↑↑). **B) 0.38 mg/kg *X. testudinaria*-treated group** with normal liver architecture. **C) 0.75 mg/kg *X. testudinaria*-treated group** with normal liver architecture (black ↑), mild dilated congested central vein (CV). **D & E) represent 1.5 mg/kg & 3 mg/ kg *X. testudinaria*-treated groups**, respectively, with marked loss of normal liver architecture, marked dilatation of the central vein (CV) and highly vacuolated hepatocytes with dark stained nuclei (black ↑). Notice the hypertrophied intra-sinusoidal cells (Von Kuepfer cells) in-between hepatocytes (yellow ↑) and areas with deeply acidophilic degenerated hepatocytes without nuclei (dot arrow). Inflammatory cells (*) around degenerated bile duct (BD). **F) Satellite group** shows the polyhedral hepatocytes arranged radially in cords around the mild congested portal vein (V) with normal vesicular basophilic nuclei and granular cytoplasm (black ↑). Some hepatocytes contain two nuclei (↑↑). Notice the thin endothelial lining of the blood sinusoids (red ↑) and Von Kupffer cells (yellow ↑). Bile duct (BD) proliferation with minimal inflammatory cell infiltration (*). H&E, Scale bar 50µm.

Evaluation of different doses of *X. testudinaria* extract on pancreatic enzymes

As shown in figure (S3 (A)), there is a significant increase in amylase level after administration of 3 mg/kg and 1.5 mg/kg of *X. testudinaria* extract compared with the control group. In contrast, the dose of 0.75 mg/kg, 0.38 mg/kg of *X. testudinaria* extract, and 3 mg/kg satellite group showed no significant changes in amylase level compared to the control group. On the same line, there are no different changes in lipase level between all tested doses of *X. testudinaria* extract compared to the control group, as shown in figure (S3 (B)).

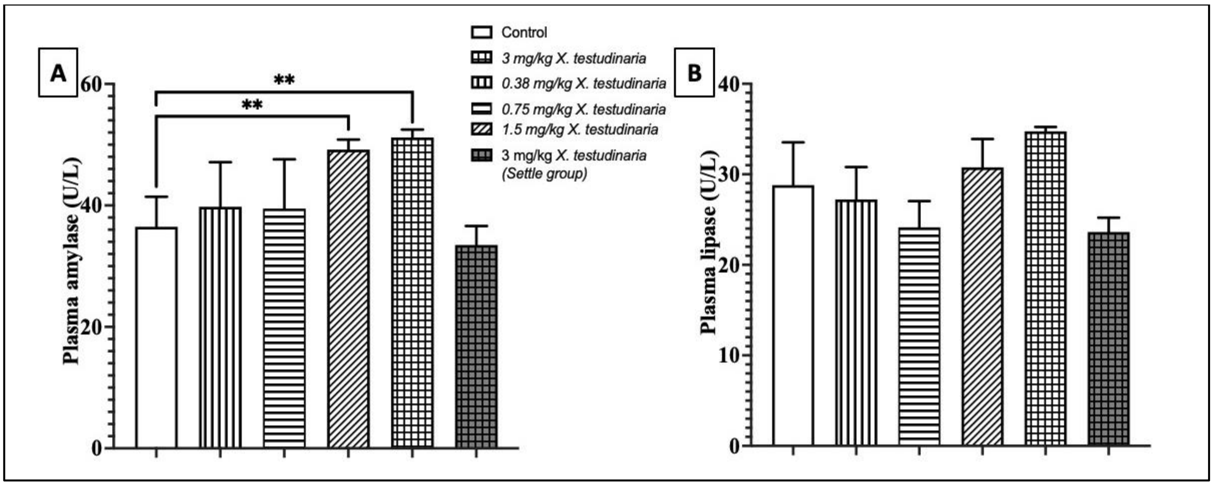

Figure S3. Evaluation of different doses of *X. testudinaria* extract on amylase (A) and lipase (B). Data are presented as mean ± SD of n=3 in each group. Statistical analysis was carried out using One-Way ANOVA. ***p*< 0.01 compared with the control group.

Evaluation of different doses of *X. testudinaria* extract on pancreas histology.

Figure S4 shows a representative section of pancreatic tissue from different treatment groups. (Figure S4 (A)) shows the normal histology architecture of pancreas of the control group. Administration of 0.38 mg/kg & 0.75 mg/kg *X. testudinaria* extract didn't change the histological architecture (Figure S4 (B & C); respectively). However, administration of 1.5 mg/kg of *X. testudinaria* extract (Figure S4 (D)) caused destruction of the normal pancreatic architecture. The blood vessels were congested and dilated with marked inflammatory cells infiltrations. The diameter of Islet of Langerhans appeared paler and decreased in size. These pathological changes were also detected with administration of the highest dose of *X. testudinaria* extract (3 mg/kg) with greater extent (Figure S4 (E)) Moreover, there is shows extensive extravasated blood RBCs with marked inflammatory cells infiltrations. However, the normal pancreatic acini and the islets of Langerhans were preserved in satellite group compared to normal group as shown in (Figure S4 (F)).

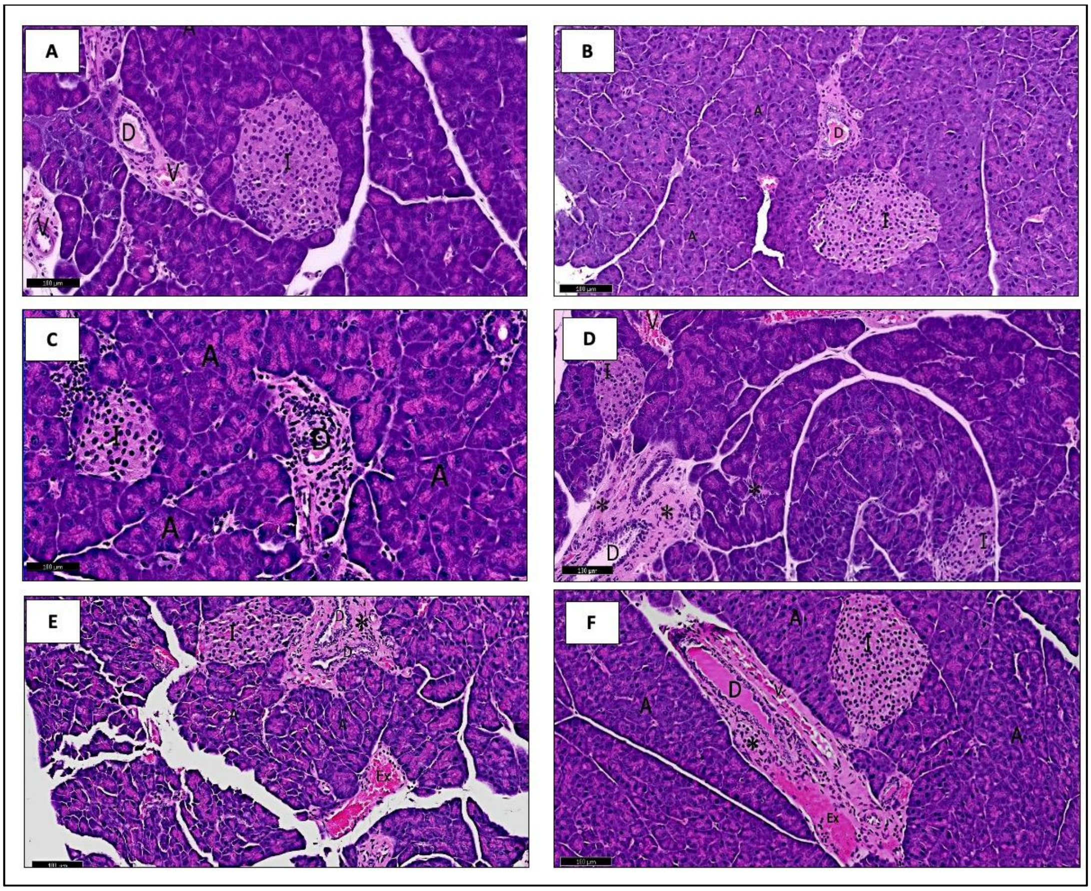

Figure S4. Effect of different doses of *X. testudinaria* extract on pancreas histology. A) control group A: control group with the normal pancreatic lobules architecture separated by thin connective tissue septa that housed blood and interlobular duct (D). An islet of Langerhans (I) surrounded by pancreatic acini (A). **B & C) 0.38 mg/kg & 0.75 mg/kg *X. testudinaria*-treated group** with normal pancreatic architecture. **D) represents 1.5 mg/kg of *X. testudinaria*-treated groups**, respectively, show destruction of the normal pancreatic architecture. Congested dilated blood vessels (v) with marked inflammatory cells infiltrations (*) and ducts (D) are seen. Apparent decrease of the diameter of the islet of Langerhans (I) and appears paler than the surrounding tissue. **E) 3 mg/ kg *X. testudinaria*-treated group** showed extensive extravasated blood RBCs (Ex) with marked inflammatory cells infiltrations (*) and ducts (D) are seen. Apparent decrease of the diameter of the islet of Langerhans (I). **F) satellite group** showed the pancreatic acini and the islets of Langerhans (I) are generally similar to the control group. Mild inflammatory cells around dilated duct (D) and mild congested blood vessels (v). Mild extravasated blood RBCs (Ex). H&E X10, Scale bar 100 µm.

Discussion

Due to the lacking of detailed evidence on the safety of IP administration of *X. testudinaria*, the acute toxicity study for the *X. testudinaria* extract was performed as an initial step to assess the harmful effect that occurs within a short time after administration of a new natural product or chemical (Hodgson, 2004). Particularly, the current study evaluated the mortality, body weight, other spontaneous change in behavior and well-being of the mice. There was no mortality reported or any toxicity signs after 24 hours of IP injection of 2000 mg/kg of *X. testudinaria* extract. Moreover, mice in the settled group showed normal behavior during the 14 days post IP injection.

For acute toxicity, the mice were given a series of single different doses *X. testudinaria* extract IP (3 mg/kg, 1.5 mg/kg, 0.75 mg/kg, 0.38 mg/kg, and 3 mg/kg settled group). However, the highest dose (3 mg/kg) showed signs of toxicity in liver histology. Administration of 3 mg/kg of *X. testudinaria* extract significantly increased serum ALP and GGT. Those enzymes are increased in case of drug-induced damage to the hepatic cells and obstruction of the bile ducts (Francis & Navarro, 2021). Interestingly, this hepatotoxicity was reversible and disappeared when the drug discontinues in the settled group. This observation has pharmacological and clinical importance. From a pharmacokinetic point of view, this observation may shed some light on the liver contribution to the *X. testudinaria* extract metabolism. Indeed, the liver is well known for its role in xenobiotic metabolism (Österreicher & Trauner, 2012). From the clinical point of view, the results of this study suggested closely mentoring the liver function during the administration of *X. testudinaria* extract. Nonetheless, further proper *in vivo* and clinical pharmacokinetic studies, including the different route of administrations, must be carried out

before concluding this assumption. In contrast, only one pervious study was done to assess oral acute toxicity for Chinese *X. testudinaria in vivo* (Liang *et al.*, 2014). Single oral administration of Chinese *X. testudinaria* up to 1600 mg/kg to the mice showed no signs of hepatoxicity and renal toxicity in the biochemical and histological analysis.

The results from this study also revealed that the dose of 1.5 & 3 mg/kg of *X. testudinaria* extract caused significant increase in pancreatic amylase, but not the pancreatic lipase, associated with signs of pancreatic toxicity in the histology. Whereas, a previous *in vitro* study showed that Chinese *X. testudinaria* significantly inhibited pancreatic enzymes (Liang *et al.*, 2014). Indeed, they proposed Chinese *X. testudinaria* as a therapeutic candidate for obesity.

Conclusion

The toxicity study revealed that IP administration of *X. testudinaria* extract did not cause any mortality or other toxicity signs, thus the LD_{50} of *X. testudinaria*is extract greater than 2000 mg/kg. Acute toxicity study suggested the doses above 1.5 mg/kg cause reversable, dose-dependent hepatotoxicity in mice shown in biochemical and histological analysis. These results provide preliminary data on the safety profile of *X. testudinaria* extract, however, further studies to investigate the pharmacokinetics of *X. testudinaria* extract are needed.
